# Japanese radiological technologists’ perceptions and interest in disaster medicine and radiation emergency medicine

**DOI:** 10.3389/fpubh.2024.1463583

**Published:** 2024-11-14

**Authors:** Takakiyo Tsujiguchi, Masato Naraoka, Tomoki Koiwa, Kanako Yamanouchi, Katsuhiro Ito

**Affiliations:** ^1^Hirosaki University, Radiation Emergency Medicine and Cooperation Promotion, Education Center for Disaster and Radiation Emergency Medicine, Hirosaki, Japan; ^2^Advance Emergency and Critical Care Center, Hirosaki University Hospital, Hirosaki, Japan; ^3^Graduate School of Health Sciences, Hirosaki University, Hirosaki, Japan

**Keywords:** radiological technologist, disaster medicine, radiation emergency medicine, medical education, nuclear disaster medical care

## Abstract

Since the Fukushima Daiichi Nuclear Power Plant accident in Japan, there has been a growing demand for radiological technologists to play active roles in radiation emergency medicine. This study administered a questionnaire survey to determine radiological technologists’ experience, interest, and confidence in disaster medicine and radiation emergency medicine as well as their educational needs, particularly regarding radiation emergency medicine. Findings showed that less than 10% of radiological technologists working at nuclear emergency core hospitals and nuclear emergency medical cooperative institutions for nuclear disaster medical care had studied disaster medicine, regardless of their affiliation, age, or years of service, and that they lacked educational experience. However, they showed interest in general disasters and emergency medicine, and all aspects necessary for the treatment of injured and sick patients in nuclear disasters, such as dosimetry and radiation control, and were willing to learn through online formats, such as e-learning, to expand their learning opportunities. This research seeks to promote a positive perception of disaster and radiation emergency medical education among radiological technologists.

## 1 Introduction

In March 2011, the Fukushima Daiichi Nuclear Power Plant (FDNPP) accident occurred in Japan following the Tohoku Pacific Ocean Earthquake. At the FDNPP, the earthquake and tsunami caused a loss of power supply, resulting in the meltdown of the reactor core, which lost its cooling function, and the release of a large amount of radionuclides from the reactor buildings (1, 2). Several medical facilities existed in the area where the evacuation order was issued; however, owing to the loss of core infrastructure and lifelines caused by the earthquake combined with the release of radionuclides, they were unable to fully perform their functions (1, 2). The release of radionuclides led to the evacuation of hospitals and older adult care facilities, placing a tremendous physical and psychological burden on evacuees due to the repeated expansion of evacuation zones and difficulties in receiving evacuees at evacuation centers (3). The medical system at the time did not anticipate that a large number of injured and sick people who may have been contaminated by radionuclides or exposed to radiation may overwhelm the healthcare system, suggesting that cooperation among related organizations was an issue in dealing with the large number of injured and sick people (4, 5).

These accidents and cases led the Japan Nuclear Regulation Authority to implement the Nuclear Emergency Response Guidelines in October 2012, and in 2015, the nuclear disaster medical care system was considerably revised to establish the current radiation emergency medical care system (6–8). At present, 24 local governments that own or are adjacent to nuclear power plants are in the process of designating “nuclear emergency core hospitals (NECH)” and “nuclear emergency medical cooperative institutions (NEMCI),” which will play a pivotal role in receiving contaminated/exposed injured patients. Furthermore, the NECH is required to have a “nuclear emergency medical assistance team (NEMAT)” to provide assistance to disaster-stricken areas in the event of a nuclear disaster. In addition to medical doctors and nurses, radiological technologists (RT) are required to register with NEMAT (9, 10).

Let us now focus on the roles and education of RT in Japan. In Japan, RTs are users of diagnostic imaging and radiation therapy equipment in clinical practice. They are also responsible for radiation safety management in each hospital. However, in nuclear disasters, in addition to knowledge of emergency medicine, knowledge of radiation protection, prevention of the spread of contamination, decontamination, and dosimetry is essential when managing patients with exposure/contaminated injuries. In addition, knowledge of disaster medicine is necessary to establish a system for dispatching or receiving support as a NEMAT. The International Atomic Energy Agency publication “Guidance for medical physicists responding to a nuclear or radiological emergency,” published in 2020, provides guidelines for professionals in the medical physics field to respond to a nuclear/radiological emergency (11). The guidelines state that it is important that national and regional plans for radiological emergencies in each country clearly define the roles and activities to be performed by medical physics specialists. Medical physics specialists, which in Japan primarily means RTs, suggests the importance of planning and education for the utilization of these very professionals in the event of a radiological emergency. However, RT training schools in Japan do not provide opportunities to learn emergency/disaster medicine as part of their pre-graduation education. It is only after the RTs are employed by medical institutions related to nuclear disasters that they learn emergency/disaster medicine related to radiation emergency medicine (REM), making the optimization of education a challenge (12). Thus, there is an urgent need to train RTs in emergency and disaster medicine in addition to radiation protection, and dosimetry.

This study investigated the current challenges and educational needs of RTs for REM education, including the extent to which RTs working for NECH and NEMCI were interested in emergency and disaster medicine related to REM as well as their learning history and confidence. Next, we analyzed the results, summarized the knowledge required for RT to be involved in REM, and offered recommendations that will contribute to the development of future educational materials.

## 2 Methods

### 2.1 Survey participants and period

RTs (*n* = 167) affiliated with 15 medical institutions in the Aomori Prefecture designated as NECH or NEMCI as of August 2023 were surveyed using a self-administered questionnaire regarding basic attributes and demand for REM education. The Aomori Prefecture was selected because it was the first local government in Japan to progressively designate an NECH, and the local government had permission to do so. To increase the collection rate, responses could be submitted by mail or online.

### 2.2 Questionnaire survey items

The questionnaire survey comprised 13 questions in total of which 8 questions were related to the basic attributes of the participants while 5 questions pertained to disaster medicine and REM. The response method was either multiple-choice, multiple-response, or open-ended, depending on the item (Table 1). The questions were developed by a group of experts consisting of medical doctors, nurses and radiological technicians who have been involved in the education of NEMAT.

**Table 1:** Questions for radiological technologists regarding disaster medicine and radiation emergency medicine.

	Question		Answer format	Answer options
Q1	What is your affiliation?		Single choice	□Nuclear emergency core hospital □Nuclear emergency medical cooperative institutions
Q2	What is the age?		Single choice	□20s □30s □40s □50s □60s
Q3	What is your gender?		Single choice	□Male □Female □No answer
Q4	How long have you worked as a radiological technologist?		Single choice	□Less than 5 years □More than 5 years, but less than 10 years □More than 10 years, but less than 15 years □More than 15 years, but less than 20 years □More than 20 years
Q5	Do you have any experience learning about disaster medicine?		Single choice	□Yes □No □I do not know or do not remember
Q6	If the answered “Yes” in Q6, please tell us the name of the disaster medicine-related training you have attended.		Free description	–
Q7	Do you have any experience learning about radiation emergency medicine?		Single choice	□Yes □No □I do not know or do not remember
Q8	If the answered “Yes” in Q6, please tell us the name of the radiation emergency medicine-related training you have attended.		Free description	–
Q9	If an exposed/contaminated patient is transported to your facility, do you feel confident that you would be able to handle the work in the emergency room (contamination examination, radiation managment, etc.).		Single choice	□I am not at all sure □Not very confident □Can not say either way □A little confident □Fairly confident
Q10	What radiation measuring instruments do you know how to use?		Multiple choices allowed	□GM survey meter □NaI(Tl) scintillation survey meter □Ionization chamber survey meter □ZnS(Ag) scintillation survey meter □Neutron survey meter □Personal dosimeter □Whole body counter □Other ()
Q11	As a radiological technologist, what would you like to learn about radiation emergency medicine?			
	11–1	How to use radiation measuring instruments	Single choice (Select one answer for each question)	□No desire to learn at all □Not very interested in learning □Do not know □Want to learn a little □Very much want to learn
	11–2	Calculation for external and internal doses of patient		
	11–3	Exposed doses to medical personnel associated with medical treatment		
	11–4	Basic emergency care flow (medical flow in the emergency room)		
	11–5	How radiological technologists move in the emergency room (e.g., timing of contamination examination intervention)		
	11–6	Fundamentals and terminology related to emergency and disaster medicine		
	11–7	Mounting and dismounting of radiation protection equipment		
	11–8	Laws and regulations concerning radiation emergency medicine		
	11–9	Methods of collecting samples for dose evaluation (blood sampling, urine sampling, etc.)		
	11–10	Preparation for receiving exposed/contaminated patients (first aid room and curing of equipment)		
	11–11	Contamination inspection and removal of curing of emergency room after medical provision to an patient		
Q12	Please tell us if there is anything else you would like to learn in addition to the items you answered in Q11.		Free description	–
Q13	Do you feel that you would be interested in utilizing radiation emergency medicine e-learning materials if they were accessible from a PC or smart phone?		Single choice	□I would like to use it very much. □I would like to make use of it □Can not say either way □I do not want to use it □Not at all

### 2.3 Statistical analysis

Fisher’s exact probability test was used to analyze the correlations between the data obtained from the questionnaire and to examine RTs’ interest in disaster medicine and REM. OriginPro 2020 (OriginLab) was used for the statistical analysis.

### 2.4 Ethical consideration

The target medical institutions and participants were informed of the purpose of the survey, the voluntary nature of responses, protection of privacy, and anonymity in writing or by email before the survey was conducted. The name of the institution was left blank when responding to the questionnaire, and consent to participate in this study was assumed to have been obtained upon submission of the questionnaire. This study was approved by the Ethics Committee of Hirosaki University Graduate School of Health Sciences (approval number: 2020–018).

## 3 Results

### 3.1 Questionnaire survey response rates and analysis of the basic attributes of participants

A questionnaire survey of 167 RTs affiliated with hospitals in Aomori Prefecture, designated as NECH and NEMCI, regarding their REM needs, resulted in 65 valid questionnaires (40.1% response rate). The results for the basic attributes of Q1–Q8 are listed in Table 2. Characteristically, even the RTs affiliated with NECH and NEMCI had few participants (9.2% of the total), indicating that they had learned about disaster medicine. Participants who reported that they had a history of learning about disaster medicine had attended the Japan Disaster Medical Assistance Team training, and none of the RTs stated that they had learned about disaster medicine in their pre-graduate education (analyzed from the free-field responses to Q6). Next, 70.8% of respondents had a history of learning about REM. In terms of the specific courses taken, all participants indicated that they had attended one of the nuclear disaster medical care training courses systematized under the leadership of the Japan Nuclear Regulation Authority after the FDNPP accident (excerpt from the free-field responses to Q8). No RTs responded that they had received REM training or disaster medical education before graduation. Incidentally, although we examined whether any differences existed due to affiliation, age, and educational attendance, no items showed significant differences (no significant differences were found when independence tests were conducted on Q1 vs. Q5/Q7 and Q2 vs. Q5/Q7).

**Table 2:** Basic attributes of participants.

Question		Answer options	Answer (%)
Q1	What is your affiliation?	□Nuclear emergency core hospital	25 (38.5)
		□Nuclear emergency medical cooperative institutions	40 (61.5)
Q2	What is the age?	□20s	15 (23.1)
		□30s	22 (33.8)
		□40s	14 (21.5)
		□50s	14 (21.5)
		□60s	0 (0)
Q3	What is your gender?	□Male	58 (89.2)
		□Female	7 (10.8)
		□No answer	0 (0)
Q4	How long have you worked as a radiological technologist?	□Less than 5 years	10 (15.4)
		□More than 5 years, but less than 10 years	12 (18.5)
		□More than 10 years, but less than 15 years	12 (18.5)
		□More than 15 years, but less than 20 years	11 (16.9)
		□More than 20 years	20 (30.8)
Q5	Do you have any experience learning about disaster medicine?	□Yes	6 (9.2)
		□No	34 (52.3)
		□I do not know or do not remember	25 (38.5)
Q7	Do you have any experience learning about radiation emergency medicine?	□Yes	46 (70.8)
		□No	11 (16.9)
		□I do not know or do not remember	8 (12.3)

### 3.2 RTs’ confidence, interest, and concern in disaster medicine and REM

In providing medical care to exposed or contaminated patients in the emergency room, RTs must contribute to contamination testing and radiation safety management. When asked if they were confident in their ability to provide appropriate interventions to the target population, 23 (35.3%) responded “very confident” or “confident” (results from Q9, partially shown in Table 3). When asked whether they were confident in their ability to use the radiation measuring instruments considered necessary to inspect the contamination of exposed/contaminated casualties and measure air dose rates when providing REM, 64 (98.5%), 63 (96.9%), and 62 (95.4%) respondents indicated that they understood how to use the Geiger-Mueller survey meters, NaI(Tl) scintillation survey meters, and pocket dosimeters, respectively. However, none of the participants could use large equipment, such as liquid scintillation counters and germanium semiconductor detectors, for external/internal exposure dose assessment using specimens from injured or sick people (Q10 results, no data posted as figures or tables). Q11 comprised 11 questions about the content of REM learning, which is considered necessary for RTs. Around 87.0% of the respondents said that they “want to learn a little” or “very much want to learn” for all items (data not shown in the chart). When asked in Q12 about additional items that they would like to learn, participants’ free-field responses indicated that they would like to learn about not only radiation control but also disaster medicine in general, such as “general knowledge necessary to work as a NEMAT” and “management of information in a disaster.” As there were no e-learning tools for RTs to learn about contamination examination techniques and radiation safety management, we asked the respondents about their expectations for such a learning format in Q13. A total of 45 (69.2%) participants responded that they would “very much like to utilize” such a tool, 13 (20.0%) responded that they “would like to utilize” such a tool, and 7 (10.8%) responded that they were “undecided.” None of the respondents answered that they did not want to use the e-learning tool (the results for Q13 are partially shown in Table 4).

**Table 3:** Results of independence tests: experience of radiation emergency medicine education vs. intervene in radiation emergency medicine.

		(Q9). If an exposed/contaminated patient is transported to your facility, do you feel confident that you would be able to handle the work in the emergency room?					
		I am not at all sure	Not very confident	Can not say either way	A little confident	Fairly confident	*p*-value
(Q7) Experience learning about radiation emergency medicine	Yes	2 (4.3%)	8 (17.4%)	14 (30.4%)	13 (28.3%)	9 (19.6%)	0.0017
	No	9 (81.8%)	2 (18.2%)	0 (0.0%)	0 (0.0%)	0 (0.0%)	
	I do not know or do not remember	3 (37.5%)	1 (12.5%)	3 (37.5%)	1 (12.5%)	0 (0.0%)	

**Table 4:** Results of independence tests: age/period of employment vs. interest in e-learning materials.

		(Q13). Do you feel that you would be interested in utilizing radiation emergency medicine e-learning materials if they were accessible from a PC or smart phone?					
		I am not at all sure	Not very confident	Can not say either way	A little confident	Fairly confident	*p*-value
(Q2) Age	20s	0 (0.0%)	0 (0.0%)	0 (0.0%)	1 (6.7%)	14 (93.3%)	0.0002
	30s	0 (0.0%)	0 (0.0%)	0 (0.0%)	4 (18.2%)	18 (81.8%)	
	40s	0 (0.0%)	0 (0.0%)	2 (14.3%)	2 (14.3%)	10 (71.4%)	
	50s	0 (0.0%)	0 (0.0%)	5 (35.7%)	6 (42.9%)	3 (21.4%)	
(Q4) Worked as a radiological technologist	Less than 5 years	0 (0.0%)	0 (0.0%)	0 (0.0%)	0 (0.0%)	10 (100.0%)	0.0062
	More than 5 years, but less than 10 years	0 (0.0%)	0 (0.0%)	0 (0.0%)	2 (16.7%)	10 (83.3%)	
	More than 10 years, but less than 15 years	0 (0.0%)	0 (0.0%)	0 (0.0%)	3 (25.0%)	9 (75.0%)	
	More than 15 years, but less than 20 years	0 (0.0%)	0 (0.0%)	1 (9.1%)	1 (9.1%)	9 (81.8%)	
	More than 20 years	0 (0.0%)	0 (0.0%)	6 (30.0%)	7 (35.0%)	7 (35.0%)	

### 3.3 Results of the independence test

Independence tests were conducted to examine the association between basic attributes and confidence, interest, interest in disaster medicine, and REM. Fisher tests were conducted for all 78 combinations of the 6 basic attributes (Q1, Q2, Q3, Q4, Q5, and Q7) and 13 questions on disaster medicine/REM (Q9, Q11-1, Q11-2, Q11-3, Q11-4, Q11-5, Q11-6, Q11-7, Q11-8, Q11-9, Q11-10, Q11-11, and Q13). Significant differences were observed for the following three combinations.

- Q7 vs. Q9
- Q2 vs. Q13
- Q4 vs. Q13

No significant differences were identified for the combinations other than those listed above. The specific results for (i) are presented in Table 3 while those for (ii) and (iii) are shown in Table 4. An association between the groups was confirmed (*p* < 0.01).

## 4 Discussion

In this study, we evaluated the interest of RTs in disaster medicine and REM, as they are key personnel in the treatment of exposed/contaminated patients during a nuclear disaster and in providing support to affected areas as NEMATs. It should be noted that this is the first survey of RTs affiliated with NECH and NEMCI, and this is the first effort to set up the questions.

The NECH and NEMCI are medical institutions that are deeply involved in medical treatment and support during nuclear disasters and were designated in each region of Japan after the FDNPP accident. Therefore, the questionnaire survey was limited to RTs affiliated with these medical institutions. Although medical institutions located near nuclear facilities should have basic knowledge of the characteristics of nuclear disasters and radiation regardless of the type of job, the purpose of this study was to investigate RTs’ interest and educational needs in terms of medical treatment and dispatch/support to narrow the focus of the study. To receive the NEMCI designation, a nuclear disaster medical cooperation organization must fulfill one or more of the seven roles specified by the Japan Nuclear Regulation Authority and not necessarily treat exposed/contaminated patients or have an NEMAT (13, 14). Therefore, we assumed that NECH RTs were more likely than NEMCI RTs to have attended trainings related to disaster and exposure medicine, but as noted in Section 3–1, no significant difference was actually found. This result may indicate that RTs have few opportunities to learn about disaster medicine; however, REM education for RTs at NECH and NEMCI has been progressing since the FDNPP accident. The role of RTs in the event of a nuclear disaster is not limited to radiation control but rather covers a wide range of matters related to disaster medicine in general, and there is a need to train RTs on disaster medicine (15).

The results in Table 3 show the correlation between confidence in interventions related to the medical treatment of exposed/contaminated patients and REM educational experiences. The specific figures reveal that no RTs had never received REM education or were unaware about it and who also answered that they were highly confident in their intervention. Thus, the results are easily understandable and show that education and training can lead to confidence in intervention in clinical practice. Recently, in light of the COVID-19 pandemic, there have been several reports in the field of REM of the use of virtual reality (VR) technology to provide teaching materials for experiencing contamination testing as well as the creation of e-learning courses that can be delivered through videos or online (16, 17). The educational effectiveness of these VR and e-learning materials was examined. Findings revealed that using the latest tools may lead to increased interest in and RT confidence in REM. Furthermore, the results in Table 4 show a correlation among interest in e-learning materials, age, and employment duration. The figures show that people of all ages and employment durations have a high level of interest in e-learning materials. This suggests that even in the field of REM, cutting-edge educational devices are gradually becoming more widely accepted. Respondents showed a wide range of interest in not only radiation-related participants, such as radiation management and dose assessment, but also basic subjects in emergency and disaster medicine, indicating that RTs working at NECH and NEMCI have a strong desire to improve themselves.

In Japan, regulations governing the content of RT education are implemented by the Ministry of Education, Culture, Sports, Science and Technology and the Ministry of Health, Labour and Welfare (18). Unfortunately, current training rules for RTs do not include terms, such as emergency medicine, disaster medicine, or REM. Based on the study findings, it can be seen that RTs working at medical institutions designated by local governments that own nuclear-related facilities show a high interest in areas, such as emergency care, disasters, and REM. Furthermore, emergency situations, such as nuclear terrorism and accidents caused by isotopes or radiation-generating devices, should be covered in medical student education. Additionally, many reports indicate that postgraduate education for working adults on REM has been improving in recent years (7, 10). Specific educational proposals include: (i) incorporate disaster medicine and radiation exposure medicine education into RT pre-graduate education, and (ii) include specific educational topics such as medical treatment of exposed/contaminated injured patients and physical biology related to dosimetry, not to mention how to use radiation measuring instruments. It should be noted that this is a self-administered survey of a small portion of Japan, but these findings contribute to the literature on disaster medicine, particularly with regard to the critical gaps in existing knowledge about RT education.

## 5 Conclusion

In conclusion, this study highlights the urgent need for enhanced education and training for radiological technologists (RTs) in disaster and radiation emergency medicine (REM) in Japan. Although RTs affiliated with nuclear emergency core hospitals (NECH) and nuclear emergency medical cooperative institutions (NEMCI) showed significant interest in REM, their pre-graduate education lacks sufficient coverage of disaster medicine. The findings underscore the importance of incorporating REM into RTs’ education and utilizing modern tools, such as e-learning and virtual reality, to improve their preparedness and confidence in managing nuclear or radiological emergencies.

## Data Availability

The original contributions presented in the study are included in the article/supplementary material, further inquiries can be directed to the corresponding author.

## References

[1] Ohtsuru A Tanigawa K Kumagai A Niwa O Takamura N Midorikawa S. Nuclear disasters and health: lessons learned, challenges, and proposals. Lancet. (2015) 386:489–97. doi: 10.1016/S0140-6736(15)60994-1, PMID: 26251394

[2] Hasegawa A Tanigawa K Ohtsuru A Yabe H Maeda M Shigemura J. Health effects of radiation and other health problems in the aftermath of nuclear accidents, with an emphasis on Fukushima. Lancet. (2015) 386:479–88. doi: 10.1016/S0140-6736(15)61106-0 26251393

[3] Yanagawa Y Miyawaki H Shimada J Morino K Satoh E Ohtomo Y. Medical evacuation of patients to other hospitals due to the Fukushima I nuclear accidents. Prehosp Disaster Med. (2011) 26:391–3. doi: 10.1017/S1049023X11006418, PMID: 22067474

[4] Morimura N Asari Y Yamaguchi Y Asanuma K Tase C Sakamoto T. Emergency/disaster medical support in the restoration project for the Fukushima nuclear power plant accident. Emerg Med J. (2013) 30:997–1002. doi: 10.1136/emermed-2012-201629, PMID: 23184925 PMC3841807

[5] Meineke V Dörr H. The Fukushima radiation accident: consequences for radiation accident medical management. Health Phys. (2012) 103:217–20. doi: 10.1097/HP.0b013e31825b5809 22951483

[6] Tsujiguchi T Sakamoto M Koiwa T Suzuki Y Ogura K Ito K. A simple survey of the preparation situation for Resident's evacuation in Japanese prefectures after the Fukushima Daiichi nuclear power plant accident. Front Public Health. (2020) 8:496716. doi: 10.3389/fpubh.2020.496716, PMID: 33123507 PMC7567010

[7] Tatsuzaki H Kumagai A Fuma S Yamashita S. Reorganization of advanced radiation emergency medicine systems in Japan after the Fukushima nuclear power plant accident. Environ Adv. (2022) 8:100197. doi: 10.1016/j.envadv.2022.100197

[8] Nagata T Arishima T Yamaguchi Y Hirohashi N Usa T Hasegawa A. Radiation emergency medical preparedness in Japan: a survey of nuclear emergency Core hospitals. Disaster Med Public Health Prep. (2022) 17:e78. doi: 10.1017/dmp.2021.348 35129102

[9] Iyama K Kakamu T Yamashita K Shimada J Tasaki O Hasegawa A. Current situation survey for establishing personally acceptable radiation dose limits for nuclear disaster responders. J Radiat Res. (2022) 63:615–9. doi: 10.1093/jrr/rrac026, PMID: 35640253 PMC9303598

[10] Tsujiguchi T Yamaguchi M Mikami J Sato D Itaki C Hosokawa Y. Survey on training of the nuclear emergency medical assistance team and their educational needs. Radiat Environ Med. (2019) 8:16–20. doi: 10.51083/radiatenvironmed.8.1_16

[11] International Atomic Energy Agency (IAEA). Guidance for medical physicists responding to a nuclear or radiological emergency. EPR-medical physicists. Vienna: IAEA (2020).

[12] Ohba T Mabune K Kanno S Hasegawa A. Recommendations for optimising a human resource development training programme for a nuclear accident based on the personal backgrounds of radiological technologists: experiences from the Fukushima Daiichi nuclear power station accident. Nihon Hoshasen Gijutsu Gakkai Zasshi. (2022) 78:1282–94. doi: 10.6009/jjrt.2022-1307, PMID: 36171114

[13] Japan Nuclear Regulation Authority. (2023). Available at:https://www.nra.go.jp/data/000359967.pdf. Accessed July 12, 2024

[14] Japan Nuclear Regulation Authority. (2022). Available at:https://www.nra.go.jp/data/000119566.pdf. Accessed July 12, 2024

[15] Yashima S Chida K. Effective risk communications through personalized consultations with pregnant women and parents by radiologic technologists after the 2011 Fukushima Daiichi nuclear disaster. Tohoku J Exp Med. (2022) 256:259–69. doi: 10.1620/tjem.2022.J001, PMID: 35264512

[16] Tomisawa T Hosokawa S Kudo H Osanai M Ota K In N. Are online simulations for radiation emergency medical preparedness less effective in teaching than face-to-face simulations? Disaster Med Public Health Prep. (2023) 17:e520. doi: 10.1017/dmp.2023.188, PMID: 37881865

[17] Tsujiguchi T Yamanouchi K Kashiwakura I. Developing an educational program to help students learn about the resident evacuation protocols and contamination inspection undertaken during nuclear disasters. Jpn J Health Phys. (2019) 54:129–34. doi: 10.5453/jhps.54.129

[18] Japan Ministry of Health, Labour and Welfare. (2022). Available at:https://www.mhlw.go.jp/content/10801000/000566738.pdf. Accessed July 12, 2024

